# Withaferin A Attenuates Angiotensin II-Induced Right Ventricular Dysfunction and Fibrosis

**DOI:** 10.3390/ijms27041877

**Published:** 2026-02-15

**Authors:** Darini Nagarajan, Vasa Vemuri, Nicholas Kratholm, Dakotah Cathey, Pranjal Sharma, Lu Cai, Jiapeng Huang, Mariusz Z. Ratajczak, Mahavir Singh, Sham S. Kakar

**Affiliations:** 1 Department of Physiology, University of Louisville, Louisville, KY 40202, USA; darini.nagarajan@louisville.edu (D.N.); nicholas.kratholm@lmunet.edu (N.K.); mahavir.singh@louisville.edu (M.S.); 2 Pediatric Research Institute, Department of Pediatrics, University of Louisville, Louisville, KY 40202, USAlu.cai@louisville.edu (L.C.); 3 Department of Pharmacology and Toxicology, University of Louisville, Louisville, KY 40202, USA; 4 Center for Integrative Environmental Health Sciences, University of Louisville, Louisville, KY 40202, USA; 5 Department of Anesthesiology and Perioperative Medicine, University of Louisville, Louisville, KY 40202, USA; 6 Department of Medicine, University of Louisville, Louisville, KY 40202, USA; 7 Brown Cancer Center, University of Louisville, Louisville, KY 40220, USA

**Keywords:** withaferin A, right ventricular dysfunction, angiotensin II, cardiac fibrosis, cardiac cachexia

## Abstract

Our previous studies have shown that continuous infusion of angiotensin II (Ang II) in C57BL/6J mice causes dysfunction and a cachexia-like pathogenesis in both skeletal muscle and the left ventricle, which is significantly reduced by withaferin A (WFA), a steroidal lactone. However, it remains unknown whether WFA can reverse right ventricular (RV) dysfunction induced by Ang II. To determine the effects of WFA in attenuating Ang II-induced RV dysfunction, we employed a model in which continuous Ang II infusion via an osmotic pump in C57BL/6J mice induced cardiac remodeling. We then focused on investigating RV performance and structural changes using echocardiography and histopathological examination, as well as quantitative real-time PCR (qRT-PCR) for mRNA expression. Echocardiographic analysis demonstrated that Ang II significantly increased RV wall thickness and impaired RV systolic and diastolic function, as indicated by reductions in tricuspid annular plane systolic excursion, TV E/E′ ratio, RV S′, and RVOT VTI. The qRT-PCR analysis revealed marked upregulation of pro-fibrotic markers, including TGF-β, fibronectin, and collagen. WFA treatment restored RV functions and significantly attenuated Ang II-induced RV dysfunction and fibrosis. Our findings provide the first evidence that WFA attenuates Ang II-induced cachexia-like remodeling and dysfunction of the RV. These results position WFA as a compelling therapeutic candidate for cardiac cachexia, offering direct anti-fibrotic and cardioprotective benefits that warrant further translational development.

## 1. Introduction

Cachexia is a multifactorial metabolic syndrome characterized by involuntary weight loss, progressive skeletal muscle wasting, adipose tissue depletion, and systemic inflammation [1,2]. It leads to functional decline, metabolic changes, and reduced survival across chronic diseases, including cancer, chronic kidney disease, and heart failure [3,4,5]. Despite its clinical relevance, cachexia remains a major unmet medical need, exerting broad metabolic and physiological consequences across multiple organ systems [6]. These systemic disturbances are further supported by biomarker profiling studies that reveal widespread alterations in circulating metabolites [7,8,9,10]. In cancer patients, cachexia frequently extends to the myocardium, resulting in cardiac cachexia marked by myocardial atrophy, fibrosis, and impaired contractility. Recent studies highlight myocardial atrophy, fibrosis, and contractile impairment as central features of cachexia-driven cardiac remodeling [11,12]. This cardiac involvement is increasingly recognized as a key prognostic determinant in almost all oncological populations [13,14]. However, current therapeutic strategies, including nutritional support and appetite stimulants, have largely failed to restore muscle mass or cardiac function, thus underscoring the urgent need to target the molecular drivers of cachexia, particularly within the myocardium [3,4,5,13].

Angiotensin II (Ang II), a major effector of the renin–angiotensin–aldosterone system, plays a central role in cardiac oxidative stress, inflammation, and remodeling via atrophy and fibrosis, and is also implicated in skeletal muscle catabolism across multiple conditions [15,16,17]. Ang II activates maladaptive pathways in cardiovascular tissues, including oxidative stress, mitochondrial disruption, inflammation, and profibrotic signaling cascades [18]. Chronic Ang II exposure suppresses anabolic signaling via inhibition of the IGF-1/AKT/mTOR pathway, increases oxidative stress through NADPH oxidase activation, and drives transforming growth factor-β (TGF-β)/SMAD-dependent fibrosis in cardiac and skeletal muscle [15,16,19]. Suppression of the IGF-1/AKT/mTOR signaling axis is a well-established mechanism underlying muscle and myocardial wasting conditions across chronic diseases [20]. Therapeutic approaches that enhance IGF-1 signaling or inhibit Ang II-mediated catabolic cascades have been shown to alleviate cachexia in multiple preclinical models [21,22,23]. Tumor-free models that recapitulate the molecular and histopathological features of cancer-associated wasting are essential for isolating direct anti-cachectic effects from tumor suppression. Continuous Ang II infusion is a well-established, tumor-independent model of cachexia-like cardiac remodeling, producing hallmark features such as myocardial fibrosis, inflammation, contractile dysfunction, and diastolic impairment [24,25]. Oxidative stress is a key driver of Ang II-induced remodeling in these models, making them well-suited for evaluating direct cardioprotective and anti-cachectic actions [26,27,28].

In this context, WFA, a steroidal lactone derived from *Withania somnifera*, has emerged as a potent anti-inflammatory and anti-cachectic agent [1,29,30,31,32,33]. WFA ameliorates cancer-induced skeletal muscle wasting and cardiac cachexia, likely through modulation of inflammatory pathways such as NF-κB and suppression of pro-cachectic gene expression in skeletal and cardiac tissues [31,34,35]. Natural bioactive compounds with anti-inflammatory or anti-fibrotic properties, including steroidal lactones like WFA, have gained attention for their ability to modulate signaling pathways relevant to cachexia pathogenesis [36,37,38]. Studies in tumor-bearing models suggest that WFA exerts both anti-tumor and anti-cachectic effects [31,35]. While left ventricular (LV) remodeling has been widely studied, right ventricular (RV) involvement is increasingly recognized as a critical determinant of clinical outcomes in heart failure and cancer cachexia. Compared with LV, the RV’s thinner wall and unique hemodynamic load make it particularly vulnerable to fibrotic stiffening, diastolic impairment, and contractile dysfunction under systemic inflammatory or neurohormonal stress [13,39]. In addition, emerging evidence indicates that RV is more prone to oxidative stress-mediated pathogenesis than LV [40,41,42,43]. Therefore, understanding the molecular drivers of RV remodeling in cachexia is essential for developing targeted cardioprotective interventions.

This study builds on our prior findings demonstrating that WFA restores LV function, reduces fibrosis by regulating profibrotic gene expression, and preserves myocardial architecture in Ang II-induced cardiac cachexia [29,34]. More importantly, despite extensive investigation of LV remodeling, the right ventricle (RV) remains understudied, even though RV dysfunction independently predicts mortality, exercise intolerance, and poor outcomes in both heart failure and cachexia, and often responds poorly to therapies optimized for LV failure [44]. The present study investigates whether WFA confers similar direct anti-cachectic and cardioprotective benefits to the RV. Using continuous Ang II infusion in C57BL/6J mice, we assessed RV function alongside profibrotic gene expression (TGF-β, fibronectin, and collagen). To the best of our knowledge, this is the first investigation of whether WFA can mitigate Ang II-induced RV fibrosis and dysfunction, providing mechanistic evidence for WFA’s direct protective effects against cachexia-like cardiac remodeling [13,16,25,31,34,35].

## 2. Results

### 2.1. WFA Restores RV Contractility and Systolic Performance

**Figure 1:**
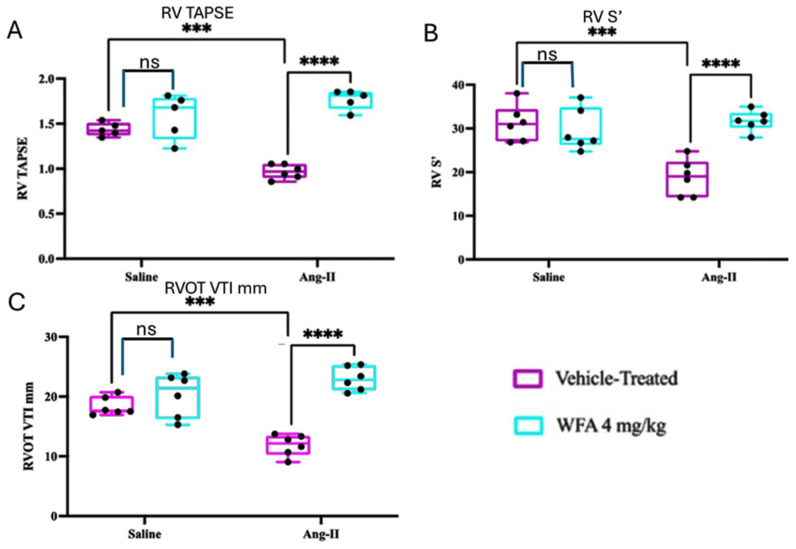
Effect of WFA on the systolic function of the right ventricle (RV). Shown are RV tricuspid annular plane systolic excursion (TAPSE, mm) (**A**), RV systolic tissue Doppler velocity S′ (cm/s) (**B**), and RV outflow tract velocity–time integral (RVOT VTI, cm) (**C**). Data shown in the boxes is median with interquartile range (N = 4–6/group). *** *p* < 0.001; **** *p* < 0.0001 by two-way ANOVA with Tukey’s post hoc test. RV = right ventricle; TAPSE = tricuspid annular plane systolic excursion; RVOT = right-ventricular outflow tract. Four animals were randomly selected from saline-infused groups and five to six from Ang II–infused groups for echocardiographic analysis. ns = non-significant.

To determine if continuous infusion of Ang II induces cardiac cachexia and impairs RV performance, and whether WFA reverses these functional deficits, we assessed RV systolic indices using transthoracic echocardiography following four weeks of Ang II infusion. Ang II-vehicle mice displayed significant RV systolic dysfunctions, including reductions in TAPSE, RV systolic tissue Doppler velocity (S′), and RVOT VTI compared with saline controls (all *p* < 0.001). Tricuspid annular plane systolic excursion (TAPSE) reflects RV longitudinal systolic shortening; systolic tissue Doppler velocity (S′) represents myocardial contractile velocity; and right ventricular outflow tract velocity–time integral (RVOT VTI) serves as an index of RV stroke distance and forward flow [45]. WFA robustly reversed these parameters. TAPSE (*p* < 0.0001) and S′ (*p* < 0.0001) were restored close to control levels, indicating recovery of RV contractility (Figure 1A,B). Likewise, RVOT VTI normalized with WFA treatment (*p* < 0.0001) (Figure 1C), demonstrating preserved forward flow and ejection performance.

Together, these results demonstrate that chronic Ang II infusion causes profound RV systolic impairment, and WFA effectively reverses these deficits. These findings complement prior reports of WFA’s protective actions in LV models, highlighting its ability to restore RV systolic mechanics and preserve output in cachexia-associated dysfunction.

### 2.2. WFA Reverses RV Structural Remodeling and Diastolic Impairment

**Figure 2:**
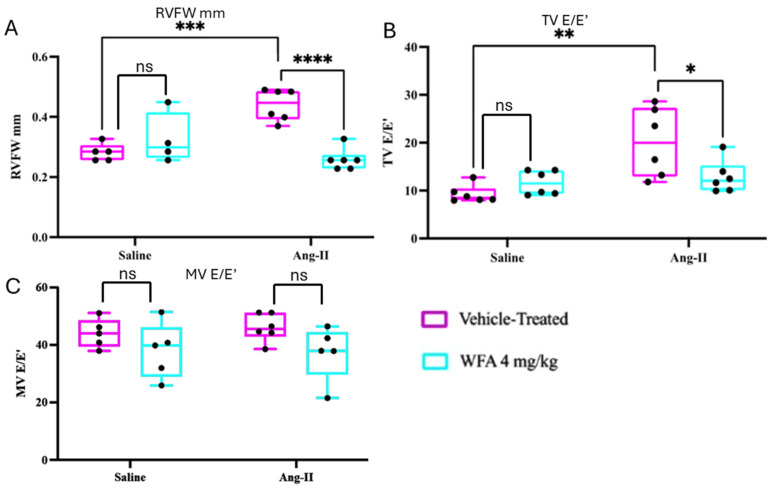
Effect of WFA on right ventricle (RV) remodeling and diastolic function in Ang II-infused mice. Shown are RV free-wall (RVFW) thickness (**A**), tricuspid valve E/E′ ratio (TV E/E′) (**B**), and mitral valve E/E′ ratio (MV E/E′) (**C**). Ang II infusion significantly increased RVFW thickness and TV E/E′ ratio, indicative of hypertrophy and elevated diastolic filling pressure, both of which were reversed by WFA. Data shown in the boxes is median with interquartile range (N = 6). * *p* < 0.05; ** *p* < 0.01; *** *p* < 0.001; **** *p* < 0.0001. ns = non-significant.

To determine whether the systolic deficits induced by Ang II were accompanied by structural remodeling, we assessed indices of RV wall structure and diastolic function. Chronic Ang II infusion promotes profibrotic and inflammatory signaling that can lead to impaired relaxation and elevated filling pressures [46,47]. Consistent with this, Ang II-vehicle mice exhibited significantly increased RV free-wall (RVFW) thickness (*p* < 0.001) and elevated tricuspid valve E/E′ (*p* < 0.001), indicating concentric remodeling and diastolic dysfunction (Figure 2A,B). Importantly, LV mitral E/E′ did not differ among groups (Figure 2C), confirming that these impairments were RV-specific.

WFA effectively reversed these maladaptive changes: RVFW thickness was markedly reduced (*p* < 0.0001), suggesting attenuation of remodeling, and TV E/E′ was normalized (*p* < 0.0001), indicating the improvement of RV relaxation. However, RV tissue Doppler E′/A′, and RV E/A ratios did not reach significance, and the LV diastolic parameters remained unchanged, thus confirming the RV-specific effects.

Collectively, these findings demonstrate that Ang II-induced cachexia triggers adverse RV remodeling and diastolic impairment, effects that are significantly mitigated by WFA. This extends findings by Vemuri et al. [29] by establishing that WFA protects RV structural integrity and filling function, independent of its tumor-related actions.

### 2.3. WFA Reverses the Cardiac Hypertrophy Induced by Ang II in the RV

**Figure 3:**
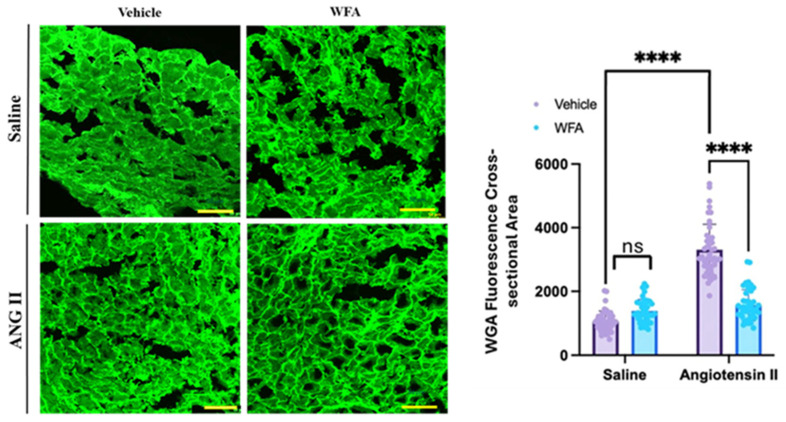
WFA attenuates Ang II-induced cardiomyocyte hypertrophy in the right ventricle (RV). Representative images of wheat germ agglutinin (WGA-stained RV sections and quantification of cardiomyocyte cross-sectional area (CSA). Cardiac tissues were stained with FITC-conjugated WGA, and four to five fields per tissue were analyzed. Fifteen cells per field were measured to determine the mean CSA. Ang II-vehicle-treated mice displayed a significant increase in CSA compared with saline vehicle controls. In contrast, WFA markedly reduced CSA toward control levels, indicating reversal of Ang II-induced hypertrophy. Data shown is mean ± SD, **** *p* < 0.0001 indicates significance. Scale bar = 50 µm. ns = non-significant.

The section above revealed the structural RV hypertrophy by echocardiography, showing WFA prevention of Ang II-increased RVFW thickness as a potential consequence of concentric hypertrophy; however, echocardiography alone cannot determine whether the wall thickening is caused by enlargement of heart muscle cells, changes in the extracellular matrix, or both. Therefore, histological analysis was needed to determine whether the myocytes are truly hypertrophic. Histological analysis using FITC–WGA staining provided independent confirmation of RV hypertrophy (Figure 3) by evaluating the size of cardiomyocytes. Quantification of the cell cross-sectional area (CSA) enclosed by the green membrane confirmed that Ang II-infused mice exhibited significantly enlarged RV myocytes CSA relative to saline controls (*p* < 0.01). WFA treatment significantly reduced CSA (*p* < 0.01), indicating that WFA prevents or reverses Ang II-induced cardiomyocyte enlargement. Representative WGA-stained sections further revealed reduced myocyte diameter and preserved architecture in the Ang II + WFA group compared with the Ang II + vehicle group. Together, these findings demonstrate that continuous Ang II infusion induces profound structural remodeling and cardiomyocyte hypertrophy in the RV, leading to compromised contractility and diastolic dysfunction. WFA reverses these maladaptive changes, normalizing wall thickness, restoring performance, and reducing cellular hypertrophy.

### 2.4. WFA Reduces the Fibrosis in Cardiac Tissue Induced by Ang II in the RV

**Figure 4:**
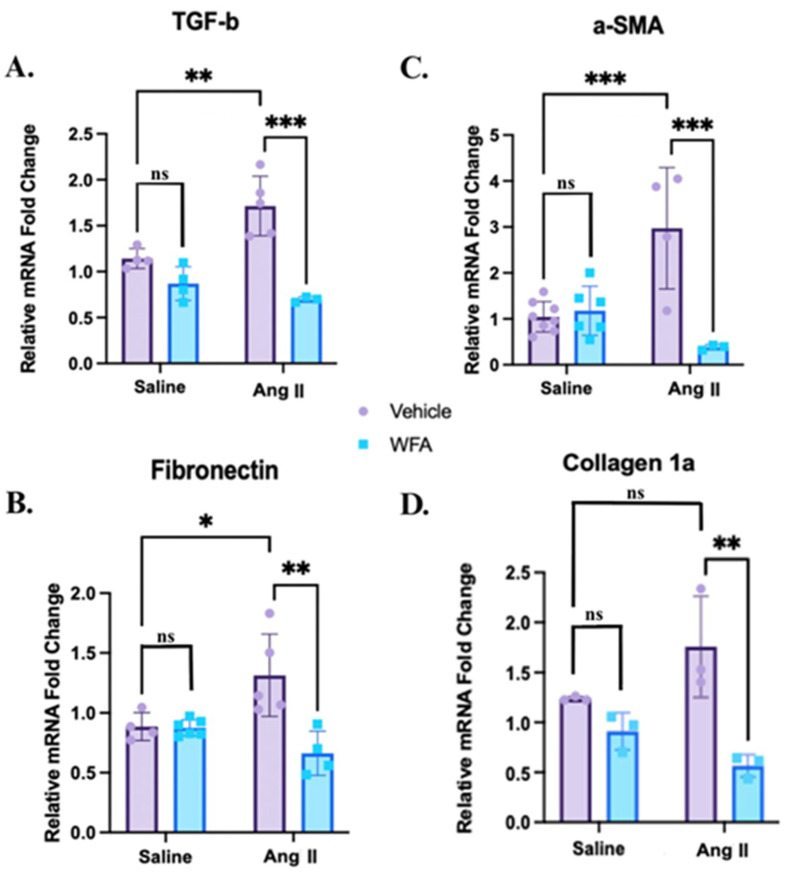
WFA attenuates Ang II-induced profibrotic gene expression in the right ventricle (RV). Mice were infused with Ang II or saline as described in Materials and Methods. RV tissue was analyzed for mRNA levels of (**A**) TGF-β, (**B**) α-SMA, (**C**) Fibronectin, and (**D**) Collagen 1a. Ang II significantly upregulated the expression of all four genes compared to saline controls, whereas WFA reduced their expression. Data are mean ± SD (n = 4–6). * *p* < 0.05; ** *p* < 0.01; *** *p* < 0.001 indicate significance. ns = non-significant.

To evaluate the effect of WFA on Ang II-induced RV fibrosis, we examined the expression of fibrosis-associated genes and histological changes in RV tissue. The qRT-PCR analysis revealed significant upregulation of TGF-β, α-SMA, Fibronectin, and Collagen 1a mRNA expression in Ang II-infused mice compared to saline controls, confirming activation of profibrotic pathways (Figure 4A–D). WFA treatment markedly attenuated expression of all four genes (*p* < 0.05 to *p* < 0.001), demonstrating that WFA effectively suppresses Ang II-induced fibrotic remodeling.

**Figure 5:**
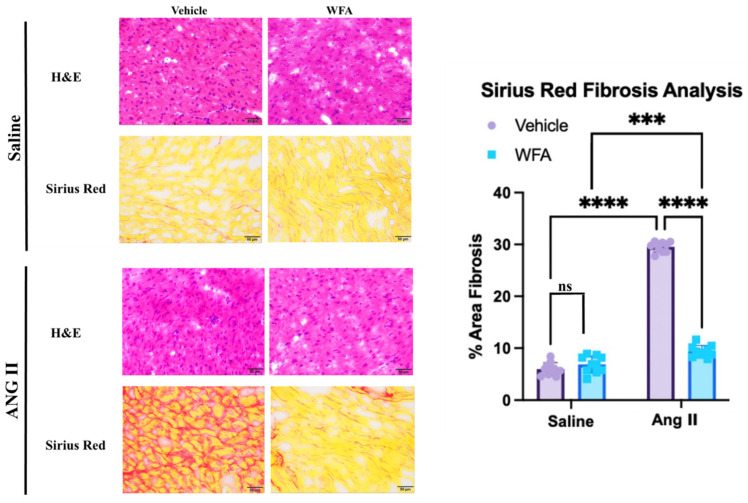
WFA inhibits Ang II-induced interstitial fibrosis in right ventricular tissue. Mice were infused with Ang II or saline as described in Materials and Methods. RV sections were stained with hematoxylin and eosin (H&E) and Sirius Red to assess fibrosis. Ang II-infusion induced substantial collagen accumulation, which was significantly reduced by WFA treatment. Quantification of Sirius Red-positive area demonstrated a significant decrease in fibrosis with WFA administration. Data shown are mean ± SD (n = 15). *** *p* < 0.001, **** *p* < 0.0001 Ang II-infused vs. saline-infused. Magnification = 40×, Scale = 50 µm. ns = non-significant.

To further support the increased expression of profibrotic genes by RT-PCR assay, histologically, Sirius Red staining also revealed increased interstitial collagen accumulation in the hearts of Ang II-vehicle mice, whereas saline controls showed minimal staining. This further supports the increased expression of profibrotic genes by RT-PCR assay. WFA treatment significantly decreased fibrotic area (*p* < 0.0001, Figure 5), confirming attenuation of extracellular matrix expansion.

These results demonstrate that WFA suppresses both transcriptional and structural markers of RV fibrosis, supporting its role as a cardioprotective anti-fibrotic therapeutic.

## 3. Discussion

The present study demonstrates that WFA effectively mitigates Ang II-induced RV remodeling and dysfunction as cachexia-like cardiac pathogenesis in a tumor-free model. Rather than merely summarizing functional outcomes, these findings are interpreted in the context of right ventricular specific susceptibility to Ang II-driven remodeling, fibrosis, and diastolic dysfunction, positioning WFA’s protective effects within established and emerging mechanistic frameworks [29]. By integrating functional, structural, and molecular assessments, our findings reveal that WFA exerts direct cardioprotective effects on the RV, a chamber whose vulnerability to stress is increasingly recognized. Importantly, this study establishes RV-specific protection by WFA, extending beyond prior LV-focused investigations and addressing a critical gap in cachexia-associated cardiac research. These results extend our previous observations in the LV [29] and establish that WFA’s benefits are not confined to a single cardiac chamber, supporting its potential as a chamber-independent anti-cachectic agent. Chronic Ang II infusion elicited a reproducible constellation of RV abnormalities characteristic of cachexia-associated cardiac remodeling, including impaired systolic function (reduced TAPSE, S′, and RVOT VTI), increased RV free-wall thickness, and elevated filling pressures, alongside activation of profibrotic gene programs [15,16,25]. These alterations align with established pathological features of cachexia-related cardiac injury, fibrosis, hypertrophy, and diminished contractility, validating the model as suitable for evaluating direct cardiac effects of therapeutic interventions independent of tumor burden.

WFA effectively ameliorated these Ang II-induced abnormalities, and the pattern of recovery provides insight into its biological significance. Functionally, WFA restored RV systolic indices toward control values, preserving longitudinal shortening and forward flow, which are critical determinants of RV performance. Structurally, WFA reduced RV free-wall thickness and normalized cardiomyocyte cross-sectional area, demonstrating reversal of hypertrophic remodeling. At the molecular level, WFA downregulated key profibrotic genes TGF-β, α-SMA, fibronectin, and collagen 1α, consistent with the observed reduction in Sirius Red-quantified collagen deposition. These molecular changes are highly relevant, as TGF-β signaling drives fibroblast activation and extracellular matrix accumulation, α-SMA reflects myofibroblast differentiation, and fibronectin and collagen Iα are central mediators of myocardial stiffening and diastolic dysfunction. Importantly, the coordinated suppression of fibrotic gene expression suggests that WFA modulates upstream remodeling programs rather than acting on isolated downstream markers [48]. In fact, TGF-β serves as a central profibrotic cytokine that drives fibroblast activation, myofibroblast differentiation, and extracellular matrix deposition in the myocardium, and α-SMA is a hallmark of activated myofibroblasts reflecting the transition of resident fibroblasts into contractile, matrix-producing cells that promote tissue stiffening. Further, fibronectin functions as an early provisional extracellular matrix scaffold that facilitates collagen assembly and amplifies profibrotic signaling, while collagen 1α represents the principal structural component of mature myocardial fibrosis responsible for increased ventricular stiffness and impaired relaxation. The coordinated suppression of these mediators by WFA, therefore, indicates broad inhibition of fibroblast activation, matrix expansion, and maladaptive RV remodeling rather than isolated gene-specific effects. Collectively, these findings indicate that WFA blocks the fibrotic–hypertrophic cycle that drives RV stiffening and dysfunction in cachexia-like states. Although upstream signaling pathways were not directly interrogated, the coordinated suppression of these profibrotic markers is consistent with established WFA actions on inflammatory and redox-sensitive signaling cascades reported in prior studies [29]. Accordingly, the present study was intentionally designed to establish phenotypic, functional, and transcriptional endpoints that justify and inform future pathway-specific investigations, including targeted interrogation of inflammatory, redox, and profibrotic signaling cascades using molecular and genetic approaches.

The physiological relevance of these findings is amplified by the unique properties of the RV. Compared with the LV, the RV is thinner-walled, more compliant, and more sensitive to afterload; consequently, fibrotic or hypertrophic remodeling disproportionately compromises its contractile reserve. Unlike the left ventricle, the right ventricle is characterized by a thinner wall, lower myocardial mass, reduced coronary perfusion reserve, and greater dependence on longitudinal fiber shortening for systolic function [49]. These structural and hemodynamic features render the RV particularly sensitive to increases in afterload, neurohormonal activation, and systemic inflammatory stress. Under conditions of Ang II excess and cachexia-like signaling, even modest fibrotic deposition or hypertrophic remodeling can disproportionately impair RV compliance, contractile reserve, and ventricular–arterial coupling, thereby accelerating functional decline compared with the LV [50]. Thus, extending WFA efficacy from the LV to the RV is clinically meaningful, as preservation of RV function remains a major unmet need in cachexia and heart failure, where RV failure frequently limits prognosis even when LV function is therapeutically supported. In this context, our demonstration that WFA improves RV systolic performance and reduces diastolic burden highlights its potential utility in conditions where RV dysfunction contributes to clinical deterioration. These RV observations and previously reported LV improvements with WFA [29] further underscore the translational promise of this therapy, capable of protecting both cardiac chambers in systemic wasting conditions. Importantly, this study also emphasizes the broader significance of targeting cachexia-related cardiac remodeling. In both heart failure and cancer cachexia, RV dysfunction is an independent predictor of mortality and poor functional outcomes [13,39]. Our data provides direct evidence that WFA addresses structural and molecular drivers of cachexia-induced cardiac injury, particularly fibrosis and hypertrophy, which represent viable therapeutic targets for preserving RV function. The cardioprotective effects observed here are mechanistically aligned with prior literature implicating inflammation, oxidative stress, and TGF-β-driven remodeling as central mediators of cachexia-associated cardiac decline, supporting WFA as a promising intervention.

This study has its limitations. First, it relies on a single Ang II-based tumor-free model, which may not fully capture the heterogeneity of human cardiac cachexia, particularly in tumor-bearing states or chronic disease conditions [24,25]. Additional validation in cancer models, alternative cachexia paradigms, and longer-term studies will be necessary to define the generalizability and durability of WFA’s protective effects. Second, while functional, histological, and transcriptional endpoints clearly demonstrate RV protection, direct interrogation of upstream signaling pathways (e.g., NF-κB, STAT3, IGF-1/AKT/mTOR, or redox regulators) was beyond the scope of this study. Targeted mechanistic studies using pathway-specific inhibitors or genetic approaches will be essential to define the molecular hierarchy of WFA action in the RV. Mechanistic pathway analyses will also be required to evaluate dose–response relationships, long-term safety, and therapeutic window [3,4,5,29,31,35]. From a translational perspective, several important considerations must be addressed before clinical application of WFA for cardiac cachexia, including optimization of dosing and formulation, characterization of pharmacokinetics and tissue bioavailability, and evaluation of long-term safety with chronic administration. In addition, validation across tumor-bearing cachexia models, diverse etiologies of systemic inflammation, and larger animal systems will be essential to define therapeutic robustness and clinical relevance. These considerations represent critical next steps toward advancing WFA from preclinical proof-of-concept to clinical translation.

**Figure 6:**
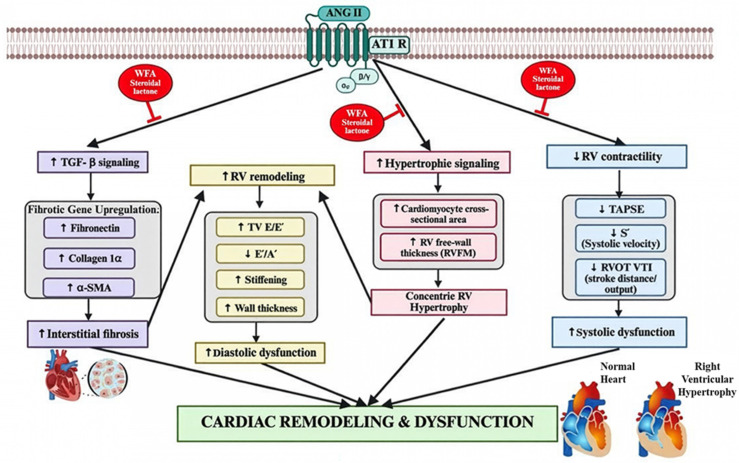
This schematic illustrates how chronic Ang II infusion drives RV dysfunction through enhanced TGF-β signaling, profibrotic gene upregulation, interstitial fibrosis, hypertrophic signaling, RV free-wall thickening, myocyte enlargement, diastolic stiffness, and impaired RV contractility (↓ TAPSE, ↓ S′, ↓ RVOT VTI). WFA ameliorates these maladaptive changes by suppressing fibrosis and hypertrophy and restoring both diastolic and systolic RV function. Collectively, WFA ameliorates Ang II-induced cardiac remodeling and improves overall RV performance. The image was created with the help of BioRender.com.

In summary, this study provides the first direct evidence that WFA counters Ang II-induced RV remodeling by restoring systolic function, normalizing diastolic indices, reversing hypertrophy, and attenuating fibrosis at both tissue and molecular levels. When considered alongside prior LV and skeletal muscle data [29], our findings suggest that WFA possesses broad, chamber-independent anti-cachectic potential (Figure 6). WFA, therefore, emerges as a promising therapeutic candidate for both cancer-associated and non-tumor cachexia in which preservation of RV function is essential for improving patient outcomes.

## 4. Materials and Methods

### 4.1. Ethical Approval

All animal experiments were conducted in strict accordance with the National Institutes of Health (NIH, Bethesda, MD, USA) guidelines for the care and use of laboratory animals. Experimental procedures were reviewed and approved by the University of Louisville Institutional Animal Care and Use Committee (IACUC) under protocol number 19653 before study initiation. The Ang II-induced cachexia model and RV assessments employed here followed standard methodologies as described previously in our published work on cachexia, each performed under approved IACUC oversight and in full alignment with NIH standards. This ethical framework, encompassing murine cachexia models, welfare safeguards, and endpoint monitoring, is consistent with established experimental practice and our group’s priority in vivo research.

### 4.2. Generation of Ang II-Induced Cachectic Mouse Model

To evaluate whether the cardioprotective effects of WFA arise from direct actions on cardiac muscle rather than secondary anti-tumor effects, we employed a well-established, tumor-independent pharmacological model of cachexia using continuous Ang II infusion, with a dose (1000 ng/kg/min) and a four-week infusion period, which was based on our prior validated studies demonstrating reproducible cachexia-like cardiac remodeling, including fibrosis and functional impairment, without inducing overt systemic toxicity or mortality [51]. Ten to 11-week-old female C57BL/6J mice (The Jackson Laboratory, Bar Harbor, ME, USA) were housed under standardized conditions at the University of Louisville animal facility. Animals were randomly assigned to receive either Ang II or sterile saline via subcutaneously implanted osmotic minipumps (Alzet Model 1004, Cupertino, CA, USA), which delivered a constant flow of 0.1 μL/hour for four weeks. Baseline body weight, forelimb strength, and total grip strength were recorded before treatment initiation. One week after pump implantation, each treatment group was further subdivided to receive intraperitoneal injections of either WFA (4 mg/kg) or vehicle control (10% DMSO, 90% glycerol trioctanoate, Sigma Aldrich, St. Louis, MO, USA) every three days, following established methods [1,52]. All experimental procedures, including cardiac functional assessments, were performed in duplicate, and data from both runs were pooled for statistical analysis. Because this project is a continuation of our LV study, the experimental design was identical to that described in Vemuri et al. [29].

### 4.3. Echocardiography and Functional Analysis of the Right Ventricle

Transthoracic echocardiography (Echo) was conducted to evaluate RV systolic and diastolic function using a high-resolution Vevo 2100 imaging system (VisualSonics Inc., Toronto, ON, Canada) equipped with a 40 MHz linear transducer probe. Mice were positioned supine on a heated platform maintained at 37 °C, with continuous monitoring of heart rate, respiratory rate, and ECG signals as previously described [39]. Anesthesia was induced with 3% isoflurane (1.5–2.5 L/min O_2_) and maintained at 1–2% throughout imaging. Anesthetic depth was verified via the absence of the toe-pinch reflex. The thoracic region was shaved, treated with depilatory cream, cleaned, and lightly coated with Neosporin ointment to prevent infection or chemical burns. A thin layer of ultrasound gel was applied to ensure optimal acoustic coupling without compression. Echocardiographic images were acquired in parasternal long-axis, parasternal short-axis, and apical four-chamber views to capture RV geometry and contractile function. Emphasis was placed on RV wall thickness, filling dynamics, and longitudinal motion to characterize changes in RV compliance and systolic performance under Ang II infusion and WFA treatment.

### 4.4. Histological Analysis of the Right Ventricle

Hearts were harvested and dissected into three components: LV, RV, and a reserved portion for biochemical analysis. Each sample was embedded in optimal cutting temperature (OCT) compound (Fisher Scientific, Waltham, MA, USA) and stored at −80 °C. Transverse cryosections (5 µm) were prepared using an Epredia Microm HM525 cryostat (Cat # 95-664-1EC, Waltham, MA, USA) maintained at −20 °C, with two sections mounted per slide for subsequent staining [53]. Hematoxylin and Eosin (H&E) staining was performed to evaluate myocardial architecture, cardiomyocyte integrity, and inflammatory changes. Sections were air-dried, fixed in 10% neutral formalin (Sigma-Aldrich Cat # HT501128, Saint Louis, MO, USA) for 10 min, rinsed in phosphate-buffered saline (PBS), stained in Harris hematoxylin (Sigma-Aldrich) for 5 min, differentiated in acid alcohol, blued in ammonia water, and counterstained with 1% Eosin Y for 2 min. Slides were dehydrated through graded ethanol, cleared in xylene, and mounted with Eukitt^®^ medium (Sigma-Aldrich Cat # 03989, St. Louis, MO, USA). This procedure provided a baseline histomorphological context for evaluation of the cardiac chambers [29,54,55]. Fibrosis was quantified using Sirius Red staining to assess interstitial and perivascular collagen deposition. Sections were fixed in Bouin’s solution (Sigma-Aldrich) for 1 h, rinsed, and stained in 0.1% Sirius Red F3B (Sigma-Aldrich, Cat # 365548) in saturated picric acid for 1 h. Slides were washed in acidified water (0.5% acetic acid), dehydrated, cleared in xylene, and mounted with Eukitt^®^. Collagen appeared red against a yellow myocardial background under brightfield microscopy. Images were captured using a calibrated digital scanner, and the fibrotic area was quantified using ImageJ (NIH, Version 1.53) with standardized thresholding [56].

Cardiomyocyte morphology was evaluated via FITC-conjugated Wheat Germ Agglutinin (WGA, Sigma Aldrich, St. Louis, MO, USA) staining to delineate sarcolemmal borders and determine cross-sectional area (CSA) [57]. Air-dried RV sections were incubated with FITC-WGA (Sigma-Aldrich, Cat # L4895; 50 µg/mL in PBS) for 45 min in the dark, then mounted using ProLong™ Diamond Antifade Mountant (Thermo Fisher Scientific, Cat # P36961, Waltham, MA, USA). Images were acquired using a Nikon Eclipse Ti confocal microscope (Nikon, Tokyo, Japan). For each section, 5–7 random fields were analyzed, and 15 cells in each field were quantified using ImageJ to calculate mean CSA, assessing myocyte hypertrophy or atrophy [54,58]. Together, these histological and morphometric analyses provided a detailed evaluation of RV remodeling, encompassing chamber morphology and cellular structure.

### 4.5. Total RNA Extraction and Quantitative Real-Time PCR

Total RNA was isolated from RV tissue using the RNeasy Fibrous Tissue Mini Kit (Qiagen, Cat # 74704, Germantown, MD, USA) according to the manufacturer’s instructions. Approximately 25–30 mg of tissue was homogenized in lysis buffer, and RNA was purified via silica-membrane spin columns. RNA concentration and purity were determined using a NanoDrop spectrophotometer (Thermo Fisher Scientific, Wilmington, DE, USA). Complementary DNA (cDNA) was synthesized from 1 µg RNA using the iScript cDNA Synthesis Kit (Bio-Rad, Cat # 170-8891, Hercules, CA, USA). Quantitative real-time PCR (qRT-PCR) was performed on a Bio-Rad CFX-Connect system using SYBR Green Master Mix and gene-specific primers (Table 1). Reactions were performed in triplicate, and melt-curve analysis verified amplification specificity. Beta-actin served as the reference gene (internal control), following previously established protocols [34,35]. Relative expression for each gene was calculated using the ΔΔCt method with efficiency correction [44,59].

### 4.6. Statistical Analysis

All analyses were conducted using GraphPad Prism version 10.0.2 (La Jolla, CA, USA). Differences among experimental groups were assessed using two-way analysis of variance (ANOVA) followed by Tukey’s multiple-comparison post hoc test. Statistical significance was accepted at *p* < 0.05 unless otherwise stated. The data are presented as mean ± SD with individual data points displayed for each group. Significance is denoted by asterisks corresponding to *p*-value thresholds, consistent with our previous reporting methods [34,35].

## Figures and Tables

**Table 1:** The sequence of the primers used.

Gene	Forward	Reverse
β-Actin	5′-CAGGCATTGCTGACAGGA TG-3′	5′-TGCTGATCCACATCTGCT GG-3′
TGF-β	5′-CTCCCGTGGCTTCTAGTGC-3′	5′-GCCTTAGTTTGGACAGGATCTG-3′
α-SMA	5′-GTCCCAGACATCAGGGAGTAA-3′	5′-TCGGATACTTCAGCGTCAGGA-3′
Fibronectin	5′-GATGTCCGAACAGCTATTTACCA-3′	5′-CCTTGCGACTTCAGCCACT-3′
Collagen-Ia	5′-GAGCGGAGAGTACTGGATCG-3′	5′-TACTCGAACGGGAATCCATC-3′

## Data Availability

All data used for statistical analysis and plotting are available upon request.

## Abbreviations

The following abbreviations are used in this manuscript:

Ang II	Angiotensin II
AT1R	Angiotensin II Type 1 Receptor
CSA	Cross-Sectional Area
ECM	Extracellular Matrix
FITC	Fluorescein Isothiocyanate
LV	Left Ventricle
NF-κB	Nuclear Factor Kappa B
Nrf2	Nuclear Factor Erythroid 2–Related Factor 2
qRT-PCR	Quantitative Real-Time Polymerase Chain Reaction
RV	Right Ventricle
RVFW	Right Ventricular Free Wall
RVOT	Right Ventricular Outflow Tract
S′	Systolic Tissue Doppler Velocity
TAPSE	Tricuspid Annular Plane Systolic Excursion
TGF-β	Transforming Growth Factor Beta
TV	Tricuspid Valve
VTI	Velocity Time Integral
WFA	Withaferin A
WGA	Wheat Germ Agglutinin
α-SMA	Alpha-Smooth Muscle Actin

## References

[1] Kakar S.S., Vemuri V., Ratajczak M.Z. (2025). Withaferin A Attenuates Muscle Cachexia Induced by Angiotensin II Through Regulating Pathways Activated by Angiotensin II. Cells.

[2] Anker M.S., Rassaf T., Zamorano J.L., Khan M.S., Landmesser U. (2024). Cardiac wasting and cancer. Eur. Heart J..

[3] Baracos V.E., Martin L., Korc M., Guttridge D.C., Fearon K.C.H. (2018). Cancer-associated cachexia—Understanding the tumour, Cancer-associated cachexia. Nat. Rev. Dis. Primers.

[4] Argilés J.M., López-Soriano F.J., Stemmler B., Busquets S. (2023). Cancer-associated cachexia—Understanding the tumour macroenvironment and microenvironment to improve management. Nat. Rev. Clin. Oncol..

[5] Fearon K.C., Glass D.J., Guttridge D.C. (2012). Cancer cachexia: Mediators, signaling, and metabolic pathways. Cell Metab..

[6] von Haehling S., Anker S.D. (2010). Cachexia as a major underestimated and unmet medical need: Facts and numbers. J. Cachexia Sarcopenia Muscle.

[7] Coêlho M.C., de Aquino G.P., Santos A.S., Seelaender M. (2024). Circulating factors in cancer cachexia: Recent opportunities for translational research. Curr. Opin. Clin. Nutr. Metab. Care.

[8] O’Connell T.M., Golzarri-Arroyo L., Pin F., Barreto R., Dickinson S.L., Couch M.E., Bonetto A. (2021). Metabolic Biomarkers for the Early Detection of Cancer Cachexia. Front. Cell Dev. Biol..

[9] More T.H., Hiller K., Seifert M., Illig T., Schmidt R., Gronauer R., von Hahn T., Weilert H., Stang A. (2024). Metabolomics analysis reveals novel serum metabolite alterations in cancer cachexia. Front. Oncol..

[10] Cao Z., Zhao K., Jose I., Hoogenraad N.J., Osellame L.D. (2021). Biomarkers for Cancer Cachexia: A Mini Review. Int. J. Mol. Sci..

[11] Wiggs M.P., Beaudry A.G., Law M.L. (2022). Cardiac Remodeling in Cancer-Induced Cachexia: Functional, Structural, and Metabolic Contributors. Cells.

[12] Tian M., Asp M.L., Nishijima Y., Belury M.A. (2011). Evidence for cardiac atrophic remodeling in cancer-induced cachexia in mice. Int. J. Oncol..

[13] Lena A., Ebner N., Coats A.J.S., Anker M.S. (2019). Cardiac cachexia: The mandate to increase clinician awareness. Curr. Opin. Support. Palliat. Care.

[14] Miyamoto Y., Hanna D.L., Zhang W., Baba H., Lenz H.J. (2016). Molecular Pathways: Cachexia Signaling-A Targeted Approach to Cancer Treatment. Clin. Cancer Res. Off. J. Am. Assoc. Cancer Res..

[15] Sukhanov S., Semprun-Prieto L., Yoshida T., Michael Tabony A., Higashi Y., Galvez S., Delafontaine P. (2011). Angiotensin II, oxidative stress and skeletal muscle wasting. Am. J. Med. Sci..

[16] Yoshida T., Delafontaine P. (2015). Mechanisms of Cachexia in Chronic Disease States. Am. J. Med. Sci..

[17] Delafontaine P., Yoshida T. (2016). The Renin-Angiotensin System and the Biology of Skeletal Muscle: Mechanisms of Muscle Wasting in Chronic Disease States. Trans. Am. Clin. Climatol. Assoc..

[18] Mehta P.K., Griendling K.K. (2007). Angiotensin II cell signaling: Physiological and pathological effects in the cardiovascular system. Am. J. Physiol. Cell Physiol..

[19] Lan X.Q., Deng C.J., Wang Q.Q., Zhao L.M., Jiao B.W., Xiang Y. (2024). The role of TGF-β signaling in muscle atrophy, sarcopenia and cancer cachexia. Gen. Comp. Endocrinol..

[20] Cohen S., Nathan J.A., Goldberg A.L. (2015). Muscle wasting in disease: Molecular mechanisms and promising therapies. Nat. Rev. Drug Discov..

[21] Song Y.H., Li Y., Du J., Mitch W.E., Rosenthal N., Delafontaine P. (2005). Muscle-specific expression of IGF-1 blocks angiotensin II-induced skeletal muscle wasting. J. Clin. Investig..

[22] Yoshida T., Semprun-Prieto L., Sukhanov S., Delafontaine P. (2010). IGF-1 prevents ANG II-induced skeletal muscle atrophy via Akt- and Foxo-dependent inhibition of the ubiquitin ligase atrogin-1 expression. Am. J. Physiol. Heart Circ. Physiol..

[23] Sacheck J.M., Ohtsuka A., McLary S.C., Goldberg A.L. (2004). IGF-I stimulates muscle growth by suppressing protein breakdown and expression of atrophy-related ubiquitin ligases, atrogin-1 and MuRF1. Am. J. Physiol. Endocrinol. Metab..

[24] Yamauchi A., Kamiyoshi A., Sakurai T., Miyazaki H., Hirano E., Lim H.S., Kaku T., Shindo T. (2019). Placental extract suppresses cardiac hypertrophy and fibrosis in an angiotensin II-induced cachexia model in mice. Heliyon.

[25] Regan J.A., Mauro A.G., Carbone S., Marchetti C., Gill R., Mezzaroma E., Valle Raleigh J., Salloum F.N., Van Tassell B.W., Abbate A. (2015). A mouse model of heart failure with preserved ejection fraction due to chronic infusion of a low subpressor dose of angiotensin II. Am. J. Physiol. Heart Circ. Physiol..

[26] Wen H., Gwathmey J.K., Xie L.H. (2012). Oxidative stress-mediated effects of angiotensin II in the cardiovascular system. World J. Hypertens..

[27] Chan S.H., Chan J.Y. (2013). Angiotensin-generated reactive oxygen species in brain and pathogenesis of cardiovascular diseases. Antioxid. Redox Signal..

[28] Du Y., Han J., Zhang H., Xu J., Jiang L., Ge W. (2019). Kaempferol Prevents Against Ang II-induced Cardiac Remodeling Through Attenuating Ang II-induced Inflammation and Oxidative Stress. J. Cardiovasc. Pharmacol..

[29] Vemuri V., Kratholm N., Nagarajan D., Cathey D., Abdelbaset-Ismail A., Tan Y., Straughn A., Cai L., Huang J., Kakar S.S. (2024). Withaferin A as a Potential Therapeutic Target for the Treatment of Angiotensin II-Induced Cardiac Cachexia. Cells.

[30] Kumar K., Bosch K., Vemuri V., Kratholm N., Rane M., Kakar S.S. (2024). Withaferin A ameliorates ovarian cancer-induced renal damage through the regulation of expression of inflammatory cytokines. J. Ovarian Res..

[31] Straughn A.R., Kakar S.S. (2019). Withaferin A ameliorates ovarian cancer-induced cachexia and proinflammatory signaling. J. Ovarian Res..

[32] Singh M., Kukreja R.C., Nagarajan D., Kakar S.S. (2025). Therapeutic potential of Withaferin A in cancer-induced muscle and cardiac wasting. J. Ovarian Res..

[33] Saha S., Singh P.K., Roy P., Vemuri V., Ratajczak M.Z., Singh M., Kakar S.S. (2025). Cancer-Induced Cardiac Dysfunction: Mechanisms, Diagnostics, and Emerging Therapeutics in the Era of Onco-Cardiology. Cancers.

[34] Kelm N.Q., Straughn A.R., Kakar S.S. (2020). Withaferin A attenuates ovarian cancer-induced cardiac cachexia. PLoS ONE.

[35] Straughn A.R., Kelm N.Q., Kakar S.S. (2021). Withaferin A and Ovarian Cancer Antagonistically Regulate Skeletal Muscle Mass. Front. Cell Dev. Biol..

[36] Vanden Berghe W. (2012). Epigenetic impact of dietary polyphenols in cancer chemoprevention: Lifelong remodeling of our epigenomes. Pharmacol. Res..

[37] Ding N., Wei B., Fu X., Wang C., Wu Y. (2020). Natural Products that Target the NLRP3 Inflammasome to Treat Fibrosis. Front. Pharmacol..

[38] Singh S., Sharma S., Sharma H. (2024). Potential Impact of Bioactive Compounds as NLRP3 Inflammasome Inhibitors: An Update. Curr. Pharm. Biotechnol..

[39] Saha S., Singh P.K., Roy P., Kakar S.S. (2022). Cardiac Cachexia: Unaddressed Aspect in Cancer Patients. Cells.

[40] Schreckenberg R., Rebelo M., Deten A., Weber M., Rohrbach S., Pipicz M., Csonka C., Ferdinandy P., Schulz R., Schlüter K.D. (2015). Specific Mechanisms Underlying Right Heart Failure: The Missing Upregulation of Superoxide Dismutase-2 and Its Decisive Role in Antioxidative Defense. Antioxid. Redox Signal.

[41] Suzuki Y.J., Shults N.V. (2017). Redox Signaling in the Right Ventricle. Adv. Exp. Med. Biol..

[42] Schwarz K., Singh S., Dawson D., Frenneaux M.P. (2013). Right ventricular function in left ventricular disease: Pathophysiology and implications. Heart Lung Circ..

[43] Stefanon I., Auxiliadora-Martins M., Vassallo D.V., Mill J.G. (1994). Analysis of right and left ventricular performance of the rat heart with chronic myocardial infarction. Braz. J. Med. Biol. Res..

[44] Raina A., Meeran T. (2018). Right Ventricular Dysfunction and Its Contribution to Morbidity and Mortality in Left Ventricular Heart Failure. Curr. Heart Fail Rep..

[45] Goldberg D.J., French B., Szwast A.L., McBride M.G., Paridon S.M., Rychik J., Mercer-Rosa L. (2016). Tricuspid annular plane systolic excursion correlates with exercise capacity in a cohort of patients with hypoplastic left heart syndrome after Fontan operation. Echocardiography.

[46] Jia G., Aroor A.R., Hill M.A., Sowers J.R. (2018). Role of Renin-Angiotensin-Aldosterone System Activation in Promoting Cardiovascular Fibrosis and Stiffness. Hypertension.

[47] Domenighetti A.A., Wang Q., Egger M., Richards S.M., Pedrazzini T., Delbridge L.M. (2005). Angiotensin II-mediated phenotypic cardiomyocyte remodeling leads to age-dependent cardiac dysfunction and failure. Hypertension.

[48] Bale S., Venkatesh P., Sunkoju M., Godugu C. (2018). An Adaptogen: Withaferin A Ameliorates in Vitro and in Vivo Pulmonary Fibrosis by Modulating the Interplay of Fibrotic, Matricelluar Proteins, and Cytokines. Front. Pharmacol..

[49] Voelkel N.F., Quaife R.A., Leinwand L.A., Barst R.J., McGoon M.D., Meldrum D.R., Dupuis J., Long C.S., Rubin L.J., Smart F.W. (2006). Right ventricular function and failure: Report of a National Heart, Lung, and Blood Institute working group on cellular and molecular mechanisms of right heart failure. Circulation.

[50] Guazzi M., Generati S.V.G., Ferraro O.E., Pellegrino M., Alfonzetti E., Labate V., Gaeta M., Sugimoto T., Bandera F. (2016). Right Ventricular Contractile Reserve and Pulmonary Circulation Uncoupling During Exercise Challenge in Heart Failure: Pathophysiology and Clinical Phenotypes. JACC Heart Fail..

[51] Fleige S., Walf V., Huch S., Prgomet C., Sehm J., Pfaffl M.W. (2006). Comparison of relative mRNA quantification models and the impact of RNA integrity in quantitative real-time RT-PCR. Biotechnol. Lett..

[52] Singh M., Pushpakumar S., Zheng Y., Smolenkova I., Akinterinwa O.E., Bana Luulay B., Tyagi S.C. (2023). Novel mechanism of the COVID-19 associated coagulopathy (CAC) and vascular thromboembolism. NPJ Viruses.

[53] Zhou H., Liu P., Guo X., Fang W., Wu C., Zhang M., Ji Z. (2024). Fibroblast-derived miR-425-5p alleviates cardiac remodelling in heart failure via inhibiting the TGF-β1/Smad signalling. J. Cell Mol. Med..

[54] Wang K., Lin Z.Q., Long B., Li J.H., Zhou J., Li P.F. (2012). Cardiac hypertrophy is positively regulated by MicroRNA miR-23a. J. Biol. Chem..

[55] Chen C., Zou L.X., Lin Q.Y., Yan X., Bi H.L., Xie X., Wang S., Wang Q.S., Zhang Y.L., Li H.H. (2019). Resveratrol as a new inhibitor of immunoproteasome prevents PTEN degradation and attenuates cardiac hypertrophy after pressure overload. Redox Biol..

[56] Zwadlo C., Schmidtmann E., Szaroszyk M., Kattih B., Froese N., Hinz H., Schmitto J.D., Widder J., Batkai S., Bähre H. (2015). Antiandrogenic therapy with finasteride attenuates cardiac hypertrophy and left ventricular dysfunction. Circulation.

[57] Emde B., Heinen A., Gödecke A., Bottermann K. (2014). Wheat germ agglutinin staining as a suitable method for detection and quantification of fibrosis in cardiac tissue after myocardial infarction. Eur. J. Histochem..

[58] Yue P., Zhang Y., Liu L., Zhou K., Xia S., Peng M., Yan H., Tang X., Chen Z., Zhang D. (2022). Yap1 modulates cardiomyocyte hypertrophy via impaired mitochondrial biogenesis in response to chronic mechanical stress overload. Theranostics.

[59] Yuan J.S., Wang D., Stewart C.N. (2008). Statistical methods for efficiency adjusted real-time PCR quantification. Biotechnol. J..

